# An Experimental Study of the Flexural Bearing Capacity of Reinforced Concrete Beams Damaged by Explosions Using Piezoelectric Smart Aggregates

**DOI:** 10.3390/s24247944

**Published:** 2024-12-12

**Authors:** Kai Xu, Shilong Sheng, Ronghui Jiang, Qian Feng

**Affiliations:** 1 School of Urban Construction, Wuhan University of Science and Technology, Wuhan 430065, China; xukai@wust.edu.cn (K.X.); sshilong@163.com (S.S.); 2 Glodon Digital Technology (Zhejiang) Co., Ltd., Hangzhou 310016, China; 18207222119@163.com; 3 School of Safety Science and Emergency Management, Wuhan University of Technology, Wuhan 430064, China

**Keywords:** piezoelectric smart aggregates, explosion load, RC beams, bending capacity, damage monitoring, wavelet packet analysis

## Abstract

Two identically sized RC beams were fabricated to investigate the effects of explosive loads on the flexural behaviour of Reinforced Concrete (RC) beams. One of the beams was subjected to an explosive load to induce post-explosion damage, and subsequently, both beams underwent flexural capacity testing. Integrating piezoelectric smart aggregates (SAas) within the beams facilitated continuous observation of the damage conditions, allowing for the assessment of internal concrete deterioration from explosive impacts to bending failures. The internal crack development index R was established using the Wavelet Packet Energy Analysis method. Combined with the structure’s residual capacity-based damage assessment criterion, the relationship between R and component damage was found. This allowed us to identify the change in the bending capacity of RC beams after explosion damage and the quantitative damage assessment of the beam to be realised, providing valuable insights for structural engineers and researchers. Comparing the flexural test results between the explosively pre-damaged beam and the undamaged test beam, it was observed that the neutral axis of the damaged beam was significantly elevated, accompanied by a notable reduction in flexural capacity. By examining the variation curve of the internal crack development index R, it was noted that during the initial stage of the bending flexural test, due to bending deformation, cracks in the core region of the pre-damaged beam gradually healed, leading to a pseudo-decrease in the damage index. After reaching a minimum point, the damage progressed faster until failure occurred.

## 1. Introduction

Reinforced concrete beams are important components in concrete structures. When concrete beams are broken, the appearance and function of the structure will be affected, thus significantly affecting the structure’s durability [1]. Explosions have a very dangerous effect on buildings as they can cause serious damage to buildings, resulting in loss of life. As blasting overpressure may cause a very strong load, beam damage caused by explosion load will shorten the service time of the structure and even cause the collapse of the structure [2]. Using effective means to monitor the damage of RC beams and study the residual bearing capacity of RC beams after blast impact damage is of great significance in evaluating the safety of RC structures.

There are a lot of studies on the analysis of antiknock performance and damage assessment of reinforced concrete beams damaged by explosion loads [3,4,5]. Yang et al. [6] studied the dynamic characteristics of reinforced concrete beams under explosion load and conducted near-field explosion tests with five different rubber-modified concrete contents. Katchalla et al. [7] adopted the LS-DYNA numerical method to establish probabilistic models of reinforced concrete beams and columns under six explosion loads. They verified the reliability of the probability equation by comparing the probabilistic models with experimental results. Li et al. [8] studied the antiknock performance of high-strength concrete beams under different concrete strengths and longitudinal reinforcement ratios. Li et al. [9] conducted explosion tests on high-strength concrete and 520 mpa stainless steel reinforcement beams to study the flexural performance of high-strength concrete beams made of stainless steel reinforcement. Xu et al. [10] studied the failure mode and dynamic response of RC beams under continuous explosions. They compared the antiknock performance of two 0.5 kgTNT continuous explosions and a single 1 kgTNT explosion at the same distance and proportional distance. Li et al. [11] conducted an explosion test on 23 P-section reinforced concrete beams with a length of 100 cm to study the effects of different explosive locations and distances on the antiknock performance of the components. TNT is located at three locations: The damage degree of reinforced concrete beams can be evaluated by observing the diameter of the crater, the spalling of concrete and the range of detonation caused by blast load. Liu et al. [12] tested ten reinforced concrete beams under different explosion conditions caused by different explosion distances and combinations of explosives. Based on the test data, an empirical expression was established to correct the relationship between mid-span displacement and proportional distance.

The bearing capacity is the basic parameter of the concrete member, and the degradation degree of the bending bearing capacity is directly related to the change in the mechanical properties of the concrete member. Residual bearing capacity is important in evaluating a component’s anti-explosion performance and damage degree. The component will lose part of its bearing capacity after experiencing an explosion load, which inevitably increases the possibility of continuous collapse of the structure. Therefore, it is very important to study the residual bearing capacity of the component after the explosion load. Ding et al. [13] studied the prediction of the residual bearing capacity of corroded RC columns based on a machine learning method, established a prediction model of the residual bearing capacity of corroded RC columns, and carried out tests on the influence of stirrup corrosion level, shear span ratio and axial load ratio on the residual bearing capacity of corroded RC columns. Liu et al. [14] took an actual fire-damaged bridge as an example, established a mechanical numerical model considering the internal temperature field, and analysed the residual bearing capacity of hollow slab beams with different damage degrees in a fire. In Chen et al. [15], to study the structural performance of corroded reinforced concrete columns under various load conditions, the regression fitting method is used to simplify the calculation of the bearing capacity coefficient of steel bars and an effective method for evaluating the residual bearing capacity of steel bars is proposed. Deng et al. [16] used nonlinear finite element analysis to study the variation in residual bearing capacity of T-shaped joints with square hollow sections under impact load. Mi et al. [17] studied the residual bearing capacity of three CFST columns and seven CFDST columns under impact loads. They analysed the effects of steel tube thickness, concrete type, impact energy and impact load on the residual bearing capacity. Molkens et al. [18] proposed an evaluation method to evaluate the residual bearing capacity of concrete structures after fire by considering the load imposed on the slabs based on the maximum allowable characteristic value of the running. Mohammed et al. [19] carried out a flexural bearing capacity test of glass fibre reinforced polymer reinforced concrete beams to study the influence of different PET fibre content concrete on the flexural performance of the components.

Various non-destructive testing (NDT) techniques can detect the state of important components in a structure. At present, the non-destructive testing methods used for building structures mainly include the ultrasonic detection method [20], the acoustic emission monitoring method [21], the image processing monitoring method [22], the optical fibre sensor [23], the impact echo method [24], etc. Kuznetsov et al. [25] detect the internal defects of concrete and reinforced concrete structures with ultrasonic low-frequency tomography instruments. Liu et al. [26] used acoustic emission technology to evaluate the mechanical properties of concrete beams strengthened by BFRP plates. They studied components’ damage process and mechanism in the flexural bearing capacity test by analysing acoustic emission signals without AE parameters. Pazdera et al. [27] used the non-destructive testing method of impact echo to determine the location and extent of internal defects in fire-damaged reinforced concrete structures. Lei et al. [28] combined UAV technology with digital image processing technology for crack detection of bridge structures. Rajabi et al. [29] used the Schmidt rebound hammer method and ultrasonic pulse velocity test as non-destructive testing to evaluate the mechanical properties of two-stage concrete (TSC) and conventional concrete.

At present, lead zirconate titanate (PZT) sensors are widely used in the field of building structural health monitoring due to their advantages such as low price [30], good piezoelectric effect [31], broadband frequency response [32], and sensing and driving functions [33,34]. However, because PZT is very fragile, it is easily affected by the external environment. Piezoelectric ceramic sheets are encapsulated in concrete blocks to be embedded in concrete structures to obtain Smart Aggregate (SA) [35]. Since then, SA has been widely used in damage monitoring, including crack monitoring of concrete structures. Li et al. [36] compared embedded SA sensors with traditional surface-mounted Acoustic emission (AE) sensors in terms of their ability to detect and assess damage to concrete structures. The results verify the feasibility of using Smart aggregates as embedded AE sensors to monitor the damage to concrete structures. Zou et al. [37] studied the influence of temperature on the performance of the SA-based active monitoring method for concrete structures. Jiang et al. [38] used an embedded piezoelectric ceramic sensor to monitor the degradation caused by corrosion in prestressed concrete structures. They verified that the proposed monitoring method can provide early warning for the initial corrosion of prestressed concrete structures. Xu et al. [39] proposed a new method for damage detection of concrete column structures under explosion load using embedded SA. They proved the effectiveness of embedded SAs active sensing in column damage monitoring under explosion load. Xu et al. [40] used SA-based active sensing technology to monitor bond slip in GFRP-reinforced concrete structures.

This paper uses the active sensing technology based on piezoelectric Smart Aggregates to conduct explosion tests on an RC beam embedded with piezoelectric Smart Aggregates. The development of cracks, the dynamic strain of concrete and the change in piezoelectric signal prove the existence of explosion damage. To study the law of the residual bearing capacity of the beam after the explosion, the bearing capacity test of the beam after the explosion test is further carried out, and the bending behaviour of the beam damaged by the blast is studied through the deformation of the beam and the change in piezoelectric signal. The internal crack development index R obtained by the wavelet packet analysis method is compared with the structural damage index D based on the damage evaluation criterion of residual bearing capacity. It is proved that piezoelectric smart aggregate sensors can effectively monitor the change in flexural bearing capacity of components after explosion damage.

## 2. Piezoelectric Smart Aggregates Monitoring Principle

### 2.1. Piezoelectric Smart Aggregates and Active Sensing Technology Loss Measurement Principle

**Figure 1:**
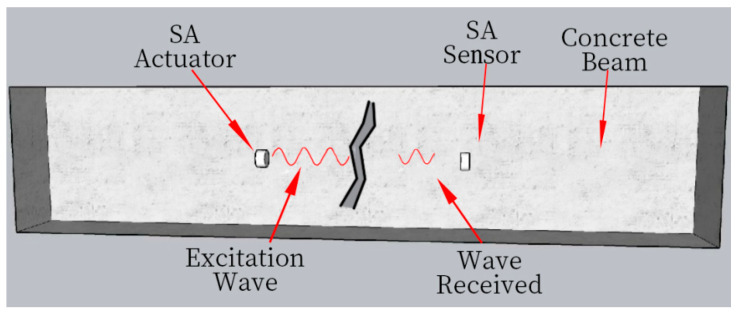
Active sensing technology damage measurement schematic diagram.

Lead zirconate titanate (PZT) is a commonly used piezoelectric material with a strong piezoelectric effect. Since PZT is a brittle material and is susceptible to the external environment, two marble blocks are used to package PZT, which can increase its service life without affecting the function of PZT material. Finally, the BNC joint is connected to form piezoelectric smart aggregates (smart aggregate, SA). From the aspects of design, materials, control, and practical application, the embedded piezoelectric smart aggregate is a reliable choice for structural monitoring in high-risk environments. Its comprehensive excellent characteristics and design advantages allow it to continue working normally even under the intense loads of an explosion [41]. SA is characterised by the piezoelectric effect, which can be divided into positive and inverse piezoelectric effects [42]. When the piezoelectric material is subjected to external load and deforms, polarisation occurs inside the material, and polarisation anomalous charge appears on the SA surface. This conversion of mechanical energy to electrical energy is called the positive piezoelectric effect. Conversely, applying an electric field to the material will create stress and strain on the material, and this conversion of electrical energy to mechanical energy is called the inverse piezoelectric effect. Then, using the piezoelectric effect of SA and the characteristics of embedding concrete structure, piezoelectric smart aggregate embedded in concrete can be used as a driver to generate stress waves or a sensor to receive stress waves. The piezoelectric smart aggregate SA1 is specified as the driving end and the aggregate SA2 as the receiving end of the signal. When the stress wave meets the damage, such as cracks in the structure during propagation, the energy of the stress wave will be attenuated, as shown in Figure 1. The monitoring signal selected was a swept sine wave with a frequency range from 100 Hz to 250 kHz, 125 k sampling points, and a signal duration of 1 s.

### 2.2. Internal Crack Development Index Based on Wavelet Packet Energy

Concrete members are those with initial cracks, and some of the energy of stress waves will be lost during transmission. However, when damage cracks appear, the stress wave energy at the receiving end will be greatly weakened, and the attenuation amplitude of stress wave energy will increase with the increase in structural damage degree. Therefore, the internal damage degree of the concrete members can be determined by comparing the stress wave energy of the piezoelectric sensor before and after damage. To avoid signal interference, it is necessary to use appropriate signal processing technology for noise reduction. Wavelet packet analysis can effectively extract the required signal amplitude. Based on multi-layer resolution analysis of the frequency band, the high-frequency signal is further decomposed into low-frequency and high-frequency signals by the Fourier transform method. The best basis function is selected adaptively to match the signal, to improve the signal analysis ability [43]. In this study, wavelet analysis was used to obtain the response value of the received energy of the sensor.

(1) $$E_{i,j}=||X_{j}|{|}_{2}^{2}=x_{j,1}^{2}+x_{j,2}^{2}+\ldots +x_{j,m}^{2}\left(j=1,2,3,\ldots ,2^{n}\right)$$

Signal S is decomposed for n times to obtain 2*n* sets of signals, where the signal in the frequency band is $X_{j}=[x_{j,1},x_{j,2},\ldots ,x_{j,m}]$(m is the sampling point). $E_{i,j}$ is time j band signal energy, and the expression is:

Therefore, the decomposed energy vector of signal S at time *i* is:

(2) $$E_{i}=[E_{j,1},E_{j,2},\ldots ,E_{j,2^{n}}]$$

Let *i* = 0; the internal structure is in a lossless state, combined with the root mean square index to determine the structural damage degree at the time *i*, and the internal crack development index *R* is the structural damage degree formula:

(3) $$R=RMSD(\%)=\sqrt{\frac{\sum_{j=1}^{2^{n}}(E_{ij}-E_{hj}{)}^{2}}{\sum_{j=1}^{2^{n}}E_{hj}^{2}}}$$

When the *R*-value approaches 0, the component is considered healthy. When the value of *R* is close to 1, the structure is in complete damage, that is, failure. When the structure is subjected to external loads, with the increase in the load, the crack’s ability to weaken the signal will directly cause the *R*-value to change from 0 to 1. Therefore, the piezoelectric active sensing technology can quantitatively evaluate and predict the damage degree inside the structure by the size of the internal crack development index *R*, which can provide theoretical guidance for the real-time dynamic health monitoring of the structure.

## 3. Fabrication of Test Components

**Figure 2:**
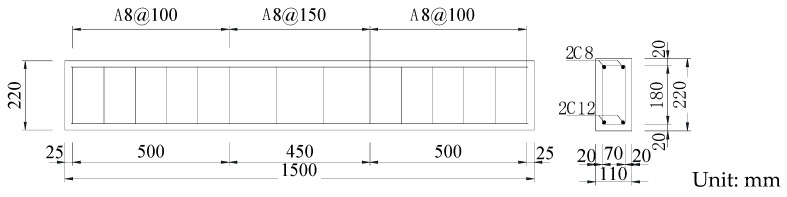
Reinforcement and geometric parameters of reinforced concrete beam.

An RC beam is designed with a double-reinforced rectangular section. The beam’s section size (b × h) is 110 mm × 220 mm, and the total length of the beam is L = 1.5 m. The layout of reinforcement bars, spacing of reinforcement bars and thickness of the protective layer of the test beam are shown in Figure 2. Before concrete is poured, two piezoelectric smart aggregates (SAs) are symmetrically fixed on the steel cage of the test beam with the span as the centre, and the distance between the two SAs is 400 mm.

**Figure 3:**
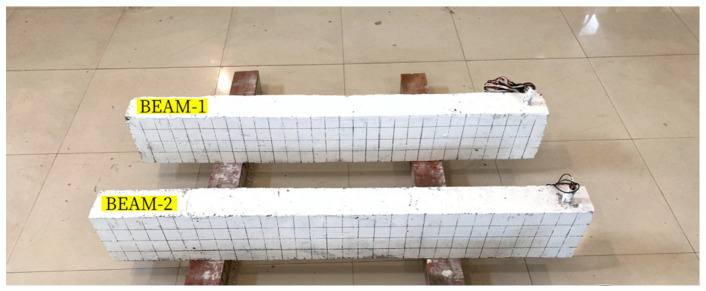
Reinforced concrete beams.

Two reinforced concrete beams were fabricated to study the flexural performance of RC beams under explosion load. The specimens are made of C40 strength-grade concrete and 42.5-grade ordinary Portland cement. The concrete mix ratio design is shown in Table 1 according to JGJ55-2011’s “Common Concrete Mix Ratio Design Regulations”[44]. After curing for 28 days under specified conditions, the cured reinforced concrete beams are shown in Figure 3, numbered Beam-1 and Beam-2, respectively.

## 4. Test Plan

### 4.1. Explosion Test on Beam-1

**Figure 4:**
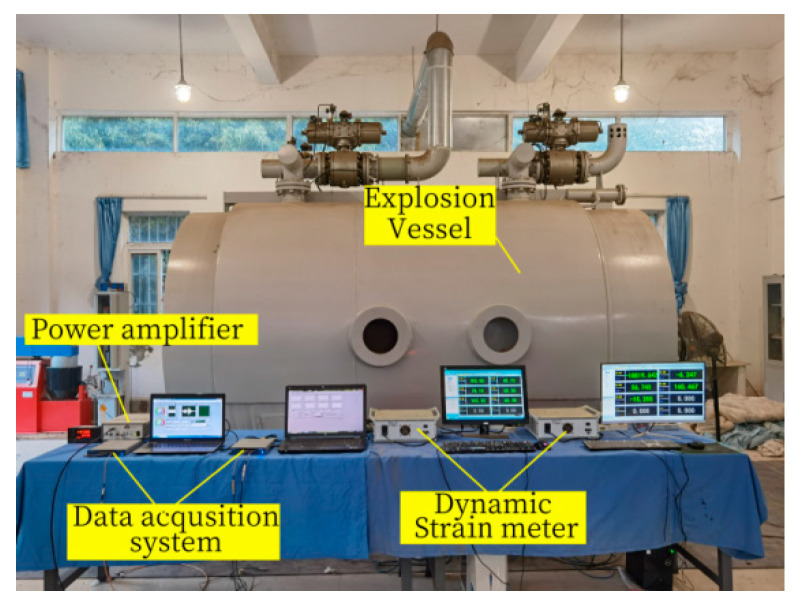
Explosion vessel and data acquisition system.

**Figure 5:**
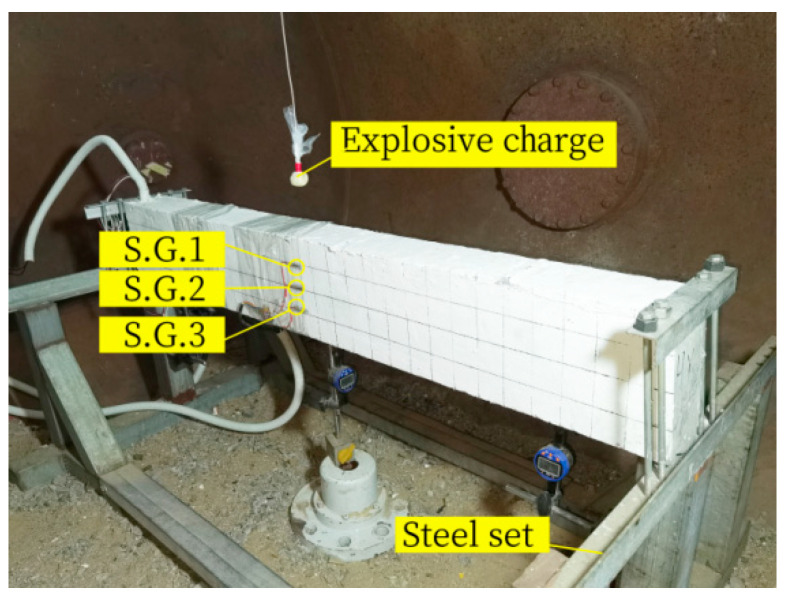
Blasting device.

To compare the flexural performance before and after the explosion load, the explosion test only causes explosion pre-damage to Beam-1, and the equipment required for the explosion test is shown in Figure 4. The explosion test was carried out in the laboratory explosion vessel. The steel support fixed the specimen beam to reasonably simulate the boundary conditions of both ends of the beam. Explosives of 100 g TNT equivalent were used in the test, and the explosives were suspended 100 mm above the centre of the detonation surface of the RC beam. A millisecond detonator was used to detonate, as shown in Figure 5. At the same time, to monitor the beam’s dynamic response, three concrete strain gauges are arranged at the side of the beam every 50 mm, numbered S.G.1, S.G.2, and S.G.3 from the top of the beam to the bottom of the beam.

### 4.2. Bearing Capacity Experiment of Two RC Beams

**Figure 6:**
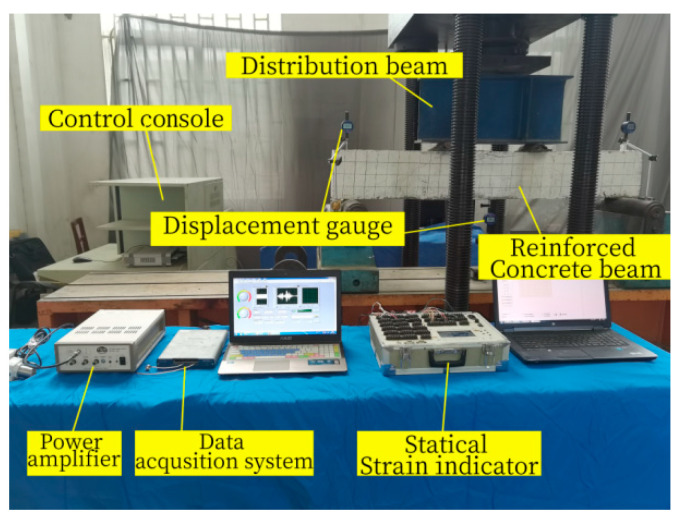
Bearing capacity test equipment layout.

**Figure 7:**
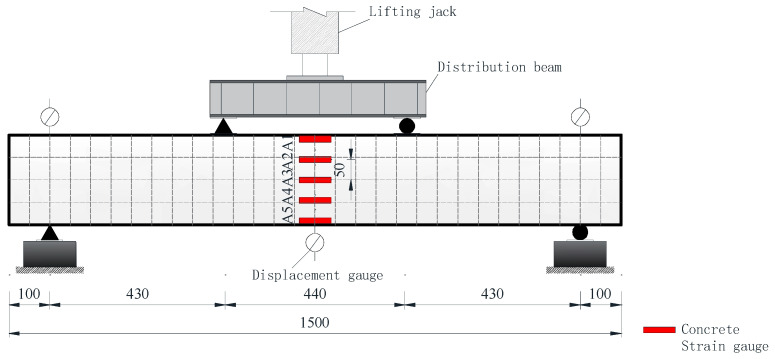
Bearing capacity test measuring point arrangement.

After the explosion test, the bearing capacity test of Beam-1 and Beam-2 is carried out. The bearing capacity test equipment is shown in Figure 6. In this test, the flexural performance is studied as four-point loading. The actuator transmits the force to the distribution beam, and then the distribution beam transmits it to the specimen beam. The transmission line connected to the actuator transmits the load data to the computer in real time. It adopts the form of hierarchical loading, and the whole loading rate is controlled at 0.1 kN/S. The load control mode is adopted in the initial stage, with each loading 5 kN. After the specimen cracks, the load of each stage is increased by 10 kN. When the load reaches 0.8 times the calculated value of ultimate bearing capacity, the load grade is encrypted, and each grade is 5 kN until the specimen is damaged. According to GBT50152-2012, the “Standard for Test Methods of Concrete structures”[45], one of the following phenomena occurs that is when the concrete in the compression zone is crushed and collapsed, the mid-span bending deflection reaches 1/5 of the span or the main crack in the tension zone reaches 1.50 mm and the beam body is judged to be damaged. Five concrete strain gauges are arranged in the beam’s side span from the top to the bottom every 50 mm to monitor the concrete strain on the beam side. The numbers are A1, A2, A3, A4 and A5 from the top to the bottom of the beam. The mid-span deformation was obtained by analysing the data change in the displacement meter, and a displacement meter was placed at the span and two supports, respectively, as shown in Figure 7.

## 5. Test Results and Analysis

### 5.1. Analysis of Explosion Damage Results of Beam-1

**Figure 8:**
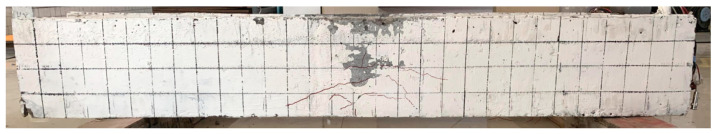
Explosion damage of Beam-1.

After the explosion test, the explosion damage of Beam-1 is shown in Figure 8. Part of the white layer on the blasting surface is washed off, and the concrete at the edge is slightly peeled off. In the middle area of the side span, there is a top-down penetrating crack and seven oblique cracks, which are roughly symmetrical distributions. The overall damage degree of the specimen beam under the action of explosion load is not large.

**Figure 9:**
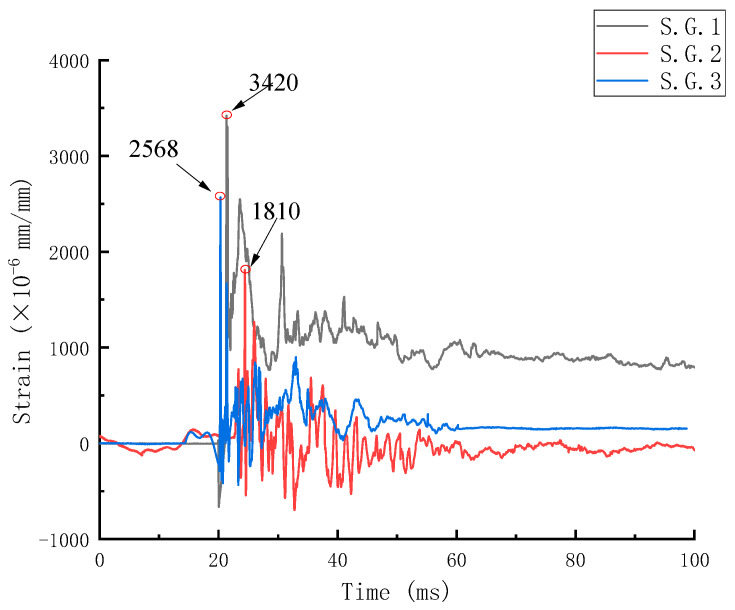
Dynamic time–history curve of concrete strain on beam side.

Figure 9 shows the time–history curve of the dynamic strain of concrete on the sides of three beams. The analysis of the results shows that, compared with the peak strain values of the three concrete strain gauges, the peak strain values of S.G.1 and S.G.2 are 1.89 times and 1.42 times those of S.G.3, respectively, and the peak strain values of concrete gradually decrease from near the explosion facing surface to near the back explosion surface. The further the distance from the explosion source, the smaller the tensile stress of the concrete and the smaller the deformation.

**Figure 10:**
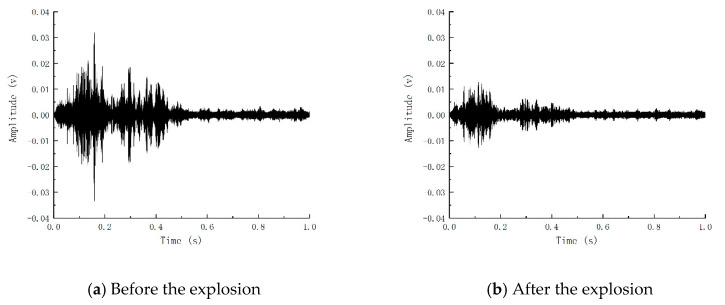
Piezoelectric signals before and after beam explosion.

Figure 10 reflects the reception of piezoelectric signals of Beam-1 before and after the explosion. It can be concluded that the blast caused damage to the components, thus weakening the piezoelectric signals. Based on the internal crack development index R obtained by wavelet packet analysis, the internal crack development index of Beam-1 is 0.79. According to the comparison of the piezoelectric signal time history curve and internal crack development index, it can be shown that the explosion load causes internal cracks and weakens the piezoelectric signal. Consistent with the damage status diagram and strain results, it reflects that the explosion load causes damage to Beam-1 and verifies that the change in piezoelectric signal obtained by piezoelectric active sensing technology can reflect the damage caused by explosion load on the member.

### 5.2. Analysis of Bearing Capacity Test Results of Two Beams

**Figure 11:**
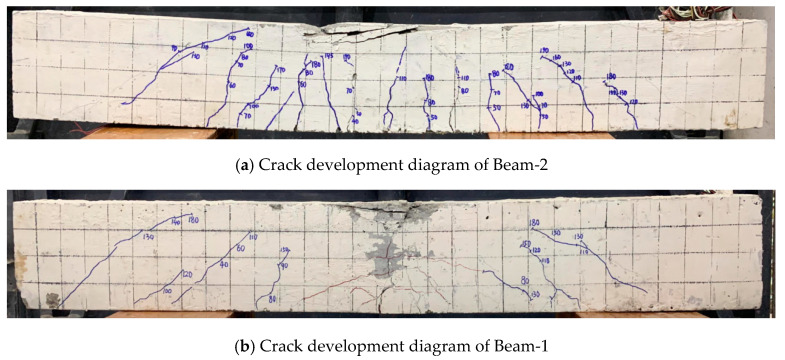
Crack development status of two beams.

Figure 11 shows the crack development of Beam-1 and Beam-2 after the bearing capacity test. By comparing Figure 11a,b, it can be seen that the number of cracks in beams without explosion damage (Beam-2) is more than that of beams with explosion damage (Beam-1) because the explosion load causes cracks in Beam-1. As the load of bearing capacity test increases, the initial crack from the explosion will develop rapidly, the concrete in the compression zone will be crushed faster, and the structure will fail. The ultimate bearing capacity of the concrete in the compression zone of beams without explosion damage is larger, which indicates that the bending bearing capacity of the beams is degraded by explosion load.

**Figure 12:**
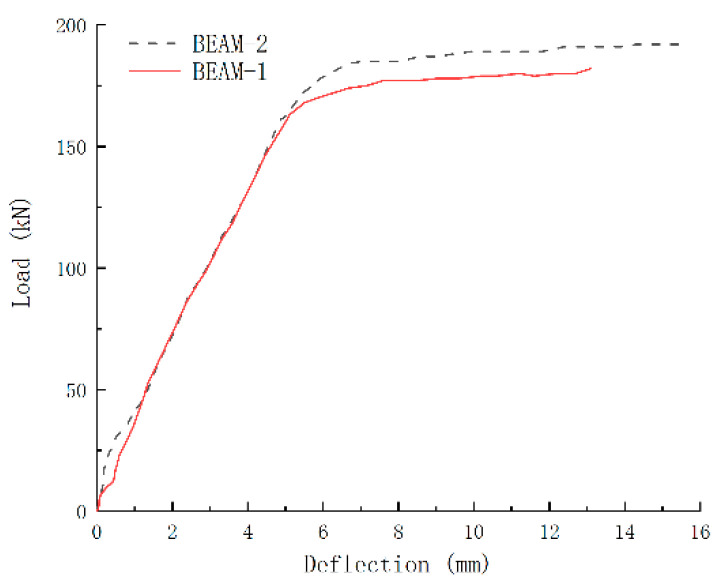
Load–deflection curve.

The load–deflection curve of the specimen beam is shown in Figure 12. The experimental results showed that the bearing capacity of the undamaged beam, Beam-2, was 197.8 kN, whereas the remaining bearing capacity of the beam Beam-1, after being subjected to an explosion, was reduced to 166.9 kN. This significant loss in bearing capacity highlights the destructive impact of explosion loads on reinforced concrete beams. By comparing the load–deflection curves of two specimen beams, it can be seen that the development law of deflection of specimen beams is similar. During the initial loading process, the mid-span deflection of Beam-1 under the same load is larger than that of Beam-2. Compared with the initial slope of the curve, it can be concluded that the initial section bending stiffness of Beam-1 is 50.3% of that of Beam-2, indicating that the explosion load weakens the initial section stiffness of the flexural member to a certain extent. In the failure stage, the final deflection of Beam-1 is reduced by 16.0% compared with Beam-2, which can prove that the flexibility of Beam-2 is better than that of Beam-1, and the plastic behaviour of reinforced concrete beam is more obvious after explosion damage.

**Figure 13:**
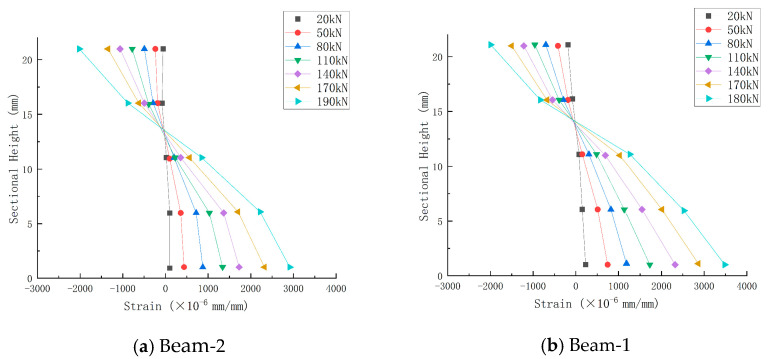
Distribution of concrete strain along section height under different loads.

The concrete strain distribution along the interface height under different loads is shown in Figure 13. From the concrete strain distribution curve of Beam-2, it can be seen that the strain distribution section of concrete at 20 kN conforms to the assumption of a plain section, and the linear relationship of strain is good. With the continuous increase in the load, the lower concrete cracks and gradually exits the work, and the position of the neutral axis begins to shift upward at 50 kN, and the relative height of the compression zone gradually decreases. At each stage of loading in the later stage, it can be observed that the height of the relative pressure zone is reduced further, and the neutral axis is moved further up until the component completely fails and is destroyed. Compared with Beam-2, it is found that the neutral axis of Beam-1 has shifted upward since the initial loading because the explosion load has caused certain damage to the entire concrete structure. Once the load is loaded, these damaged micro-cracks will quickly go through and crack, resulting in the neutral axis’s upward movement and the compression zone’s reduction during the initial loading process.

**Figure 14:**
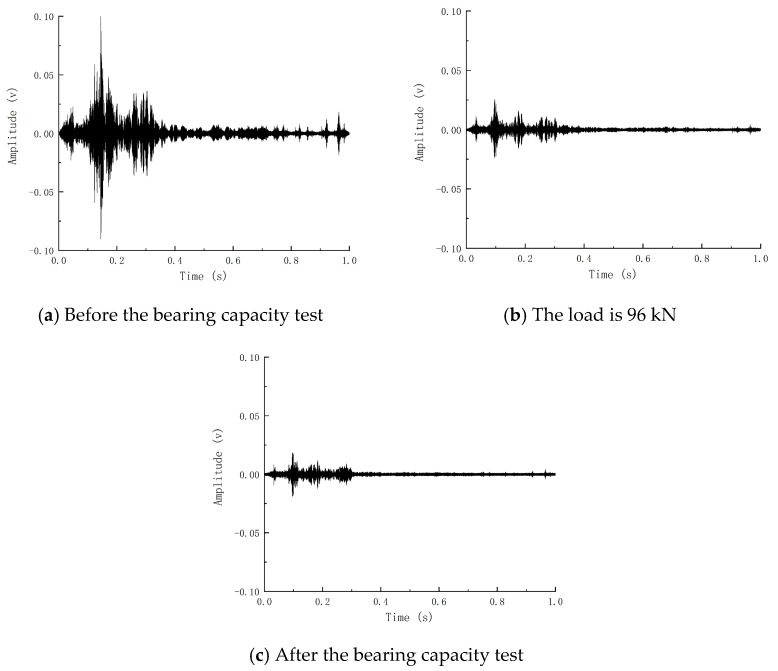
Piezoelectric signal of beam bearing capacity test of Beam-2.

**Figure 15:**
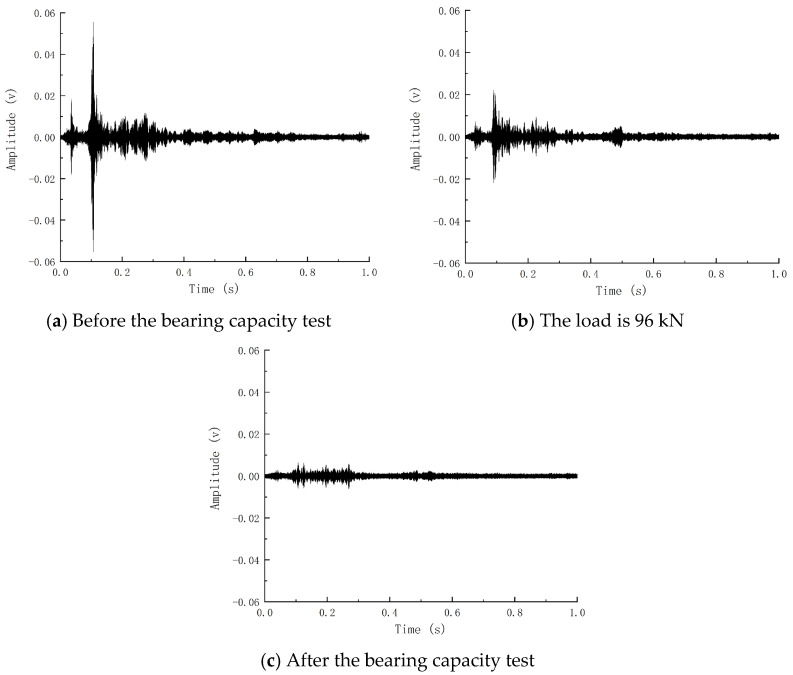
Piezoelectric signal of beam-1 bearing capacity test.

Figure 14 shows the time history curve of the piezoelectric signal waveform before and after the bearing capacity of Beam-2. It can be seen that the signal before the bearing capacity is fuller, and the piezoelectric signal gradually weakens with the increase in load. In contrast, the signal after the bending failure of the member is greatly reduced in terms of amplitude and energy, indicating that the member’s failure will significantly weaken the piezoelectric signal. The signal waveform time history curve of Beam-1 before and after static pressure is shown in Figure 15. The development of the piezoelectric signal of Beam-1 is different from that of Beam-2. Before static pressure, the receiving end of Beam-1 can still receive a certain intensity signal. The signal is enhanced because the concrete microcracks in the compression zone created by the explosion are constantly squeezed and closed during load increase. To a certain extent, the cracks in the concrete are reduced, and the piezoelectric signal is strengthened. After that, the signal began to decline rapidly until the component was destroyed, and the signal disappeared.

**Figure 16:**
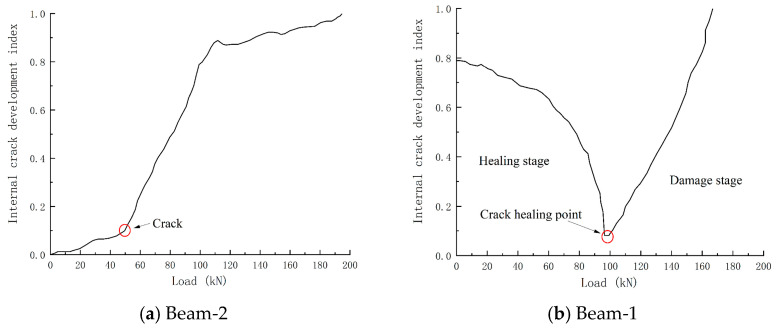
Internal crack development index-load curve.

After the piezoelectric signal was processed by the wavelet packet analysis method, it was converted into the internal crack development index-load curve under continuous loading during the test process, as shown in Figure 16. The damage curve of Beam-2 is regarded as three stages: the beam is healthy before static pressure, and the internal crack development index changes gently in the initial stage, when the beam is in the elastic stage. After reaching the cracking load, the internal crack development index has a relatively obvious steep decline, called the crack development stage. When the generation and development of cracks reach a certain extent, it is mainly due to the expansion of crack width. The growth rate of the internal crack development index tends to be flat until the bearing capacity of the member is completely lost. The damage index becomes 1, and the signal of Beam-1 is different from that of Beam-2. The damage development of Beam-1 can be regarded as two stages: the healing and failure sections. The crack development index in the healing section will gradually decrease to 0. The crack development index in the failure section will rapidly develop to 1. The turning point of the crack development index is defined as the crack healing point, and the appearance of the crack healing point corresponds to the piezoelectric signal amplitude increasing to the maximum immediately. From the internal crack development curve, it can be seen that the crack healing point of Beam-1 appears at 96 kN, and its residual flexural bearing capacity is 166.9 kN. In the test, the appearance of the crack healing point means that 57.52% of the ultimate load has been reached, and the appearance of the crack healing point means that the internal damage of the member will develop rapidly until the failure of the member. The fracture healing point of the damaged component can predict the damage of the component in advance and give a warning.

### 5.3. Analysis of Flexural Capacity

As a basic parameter of reinforced concrete beams, flexural capacity is directly related to the damage of reinforced concrete beams and can be used to evaluate various failure modes. Therefore, flexural capacity is an important parameter for damage assessment of reinforced concrete beams. The damage assessment method based on residual bearing capacity [46] is used to quantitatively evaluate the damage of reinforced concrete beams under explosion load. The expression of damage index *D* is defined as follows:

(4) D=1−N’/N

*N*’ is the residual bearing capacity of beams after the explosion, and *N* is that of unexploded damaged beams. The damage grade of reinforced concrete beams based on bearing capacity degradation is defined as follows: *D* = 0.0~0.2, indicating mild injury; *D* = 0.2~0.5, indicating moderate injury; *D* = 0.5~0.8, indicating serious injury; *D* = 0.8~1.0, indicating collapse.

Currently, the corresponding *R*-value is 0.79, reflecting the beam’s local damage. Specifically, it is the internal damage of the concrete in the core area and is also where the blast load acts. Therefore, compared with the global damage, the damage is more obvious. This shows that the piezoelectric active sensing technology can sensitively capture the structure’s damage and load-bearing capacity reduction. In addition, the piezoelectric signal is very sensitive to the structure’s cracking, so its internal damage index is relatively larger than the degradation of the load-bearing capacity, which can provide a good early warning function for engineering structures under sudden load.

Combining the piezoelectric signal and the residual bearing capacity damage index, the deterioration of the component bearing capacity can be accurately monitored using piezoelectric active sensing technology. This highlights the importance of incorporating this technology in structural health monitoring systems for improved maintenance and safety.

## 6. Conclusions

(1) Based on piezoelectric active sensing technology, the internal crack development index was defined to evaluate the internal damage degree of RC beams, and the dynamic damage process of specimen beams under explosion load was tracked. The results show that the internal crack development index of RC beams subjected to explosion load increases significantly, which proves that the piezoelectric active sensing technology can effectively monitor the dynamic damage of RC beams under explosion loads.

(2) In the bending test, the undamaged beam’s internal crack development index can be divided into three stages: initial, development, and failure. A pre-expositional damaged beam has two stages: healing and destruction. When the static pressure load reaches 143 kN, the crack healing point appears, and 78.37% of the ultimate load of the member has been reached. It indicates that the fracture healing point is close to the ultimate load of the member failure, and the fracture healing point of the damaged member can predict the member’s failure in advance and give a warning.

(3) After the built-in piezoelectric smart aggregates detect the explosion damage, the static load test is conducted to verify the results. The results show that the internal crack development index of the RC beam under the explosion load increases while its residual bending capacity decreases, showing mild damage. This proves the effectiveness of the piezoelectric active sensing technology for monitoring the change in the bearing capacity of the member.

## Figures and Tables

**Table 1:** Concrete mix ratio.

Type	Water-Cement Ratio (%)	Cement (kg/m^3^)	Sand (kg/m^3^)	Stone (kg/m^3^)	Water (kg/m^3^)
C40	0.39	432	558	1242	168

## Data Availability

The data is unavailable due to privacy restrictions. However, it can be made available upon reasonable request by contacting the author.
